# LysoTracker is a marker of differentiated alveolar type II cells

**DOI:** 10.1186/1465-9921-14-123

**Published:** 2013-11-11

**Authors:** Joanne L Van der Velden, Ivan Bertoncello, Jonathan L McQualter

**Affiliations:** 1 Lung Health Research Centre, Department of Pharmacology and Therapeutics, University of Melbourne, Melbourne, Victoria, Australia

**Keywords:** Alveolar type II cells, LysoTracker, Lung, Differentiation, Cell culture, Flow Cytometry

## Abstract

**Background:**

LysoTracker Green DND-26 is a fluorescent dye that stains acidic compartments in live cells and has been shown to selectively accumulate in lamellar bodies in alveolar type II (AT2) cells in the lung. The aim of this study was to determine whether the accumulation of LysoTracker in lamellar bodies can be used to isolate viable AT2 cells by flow cytometry and track their differentiation in live-cell culture by microscopy.

**Methods:**

Mouse lung cells were sorted on the basis of CD45^neg^CD31^neg^EpCAM^pos^LysoTracker^pos^ expression and characterized by immunostaining for SP-C and cultured in a three-dimensional epithelial colony-forming unit (CFU-Epi) assay. To track AT2 cell differentiation, lung epithelial stem and progenitor cells were cultured in a CFU-Epi assay with LysoTracker-supplemented media.

**Results:**

The purity of sorted AT2 cells as determined by SP-C staining was 97.4% and viability was 85.3%. LysoTracker^pos^ AT2 cells generated SP-C^pos^ alveolar epithelial cell colonies in culture, and when added to the CFU-Epi culture medium, LysoTracker marked the differentiation of stem/progenitor-derived AT2 cells.

**Conclusions:**

This study describes a novel method for isolating AT2 cells from mouse lungs. The high purity and viability of cells attained by this method, makes them suitable for functional analysis *in vitro*. The application of LysoTracker to live cell cultures will allow better assessment of the cellular and molecular mechanisms that regulate AT2 cell differentiation.

## Background

Alveolar type II (AT2) cells are specialized epithelial cells in the lung and comprise the majority of cells in the alveoli. They are responsible for the production and secretion of lung surfactant and can also give rise to alveolar type I (AT1) cells during development [1] and following injury in the adult lung [2].

Given the importance of AT2 cells in surfactant secretion and their role in the maintenance of alveolar homeostasis, reliable methods for their isolation and characterization in vitro are highly desirable. Many strategies have been developed for the isolation of AT2 cells for molecular analysis and functional cell culture studies. The most widely used is a modification of a method first described by Dobbs and colleagues [3], in which AT2 cells are isolated from disaggregated lung tissue by IgG-panning and immunomagnetic leukocyte depletion. Recently, a number of investigators have developed protocols to enrich AT2 cells using flow cytometry on the basis of negative antibody-labeling [4,5]. While high cell purities of between 90 to 95% have been achieved using panning and flow cytometry techniques [5-7], these isolation methods rely on negative-selection and there is considerable variability in the yield and purity reported between groups. Recent studies have identified CD74 as a marker for positive selection [8]. However, the relatively low expression of this marker does not allow the complete resolution of this population from other epithelial types.

Here, we report a novel method for isolating AT2 cells on the basis of positive LysoTracker Green DND-26 staining. LysoTracker is a fluorescent dye that stains acidic compartments in live cells. It has been previously shown to selectively label lamellar bodies in cultured mouse and rat AT2 cells [9,10]. In the present study we show that viable primary AT2 cells can be isolated to high purity on the basis of LysoTracker staining and that LysoTracker is a useful marker of AT2 cell differentiation *in vitro*.

## Methods

### Mice

Female C57Bl/6 mice (6–9 weeks age), were maintained in compliance with the Australian Code of Practice for the Care and Use of Animals for Scientific Purposes and had free access to food and water. Experiments were approved by the Animal Ethics Committee of the University of Melbourne.

### Lung cell preparations and flow cytometry

Single cell suspensions of lung cells were prepared as previously described [11] with minor modifications. Lungs were minced with a razor blade and incubated with Liberase (1 Wuncsh; Roche) in Hank’s buffered saline solution (HBSS; Invitrogen) in a volume of 4 mL/lung for 45 min at 37°C in a shaking incubator. Cells were then washed with HBSS plus 2% fetal bovine serum (FBS; Invitrogen) and resuspended in a red blood cell lysis buffer (10 mM KHCO_3_, 150 mM NH_4_Cl, 0.1 mM EDTA-Na_2_, pH 7.4) for 90 sec at room temperature. Cells were filtered through a 40 μm nylon net strainer, washed and resuspended in Dulbecco’s Modified Eagle’s Medium/F12 (DMEM/F12; Invitrogen) containing LysoTracker Green DND-26 (Invitrogen) at 37°C for 45 mins. Cells were washed and resuspended in a cocktail of fluorescein-conjugated antibodies including rat anti-mouse CD45, CD31 and EpCAM (Biolegend) and incubated on ice for 20 mins. Labeled cells were washed in HBSS plus 2% FBS and resuspended in 1 μg/mL propidium iodide (PI; Invitrogen) for flow cytometry. Viability was determined by staining unfixed cells with propidium iodine (PI), which is an intercalating agent that is unable to permeate live cells but can penetrate the porous cell membranes of dying or dead cells. Doublets were excluded by forward/side scatter-height vs. forward/side scatter-width gating. Sorting was performed with a BD FACS Aria III Cell Sorter using a 100 μm nozzle at 30 psi. Analysis was performed with a BD LSR Fortessa analyzer. Data was analyzed using FlowJo vX (Tree Star) software. Setting of LysoTracker^pos^ gates was based on full staining minus LysoTracker controls (Additional file 1: Figure S1).

Viable AT2 cells were sorted on the basis of their PI^neg^ CD45^neg^ CD31^neg^ EpCAM^pos^ LysoTracker^pos^ staining characteristics, and lung epithelial stem/progenitor cells were isolated on the basis of their PI^neg^ CD45^neg^ CD31^neg^ EpCAM^pos^ CD24^low^ signature profile as previously described [11,12].

### Epithelial colony-forming unit (CFU-Epi) assay

Sorted epithelial cells (5 ×10^4^ AT2 cells/mL or 1×10^4^ epithelial progenitor cells/mL) were mixed with Mlg cells (Murine lung fibroblast cell line; American Type Culture Collection CCL-206) (2 × 10^6^ cells/mL) and resuspended in 25 μL of Matrigel pre-diluted 1:1 (v/v) in DMEM/F12 containing 10% fetal bovine serum (FBS), penicillin, streptomycin, glutamax (PSG; Invitrogen), insulin, transferrin and selenium (ITS; Invitrogen), and 0.0002% heparin (Stem Cell Technologies). Three replicate 25 μL droplets of this cell suspension were deposited on the surface of individual 6-well Transwell filter inserts (Millicell-CM; Millipore) placed in wells containing 1.2 mL of DMEM/F12 supplemented with 10% FBS, PSG, ITS and heparin. Airway, alveolar and mixed-lineage epithelial colonies were defined by their distinct morphologies as previously described [11].

### Immunohistochemistry

Cytospots of isolated CD45^neg^CD31^neg^EpCAM^pos^LysoTracker^pos^ cells and whole-mount cultures were fixed in 4% paraformaldehyde and labeled using standard immunohistochemistry techniques. AT2 cells were labeled with rabbit anti-proSP-C (1/100; Millipore) overnight at 4°C. Rabbit IgG (Millipore) was used as an isotype control. After washing, cells were incubated with Alexa Fluor-568 conjugated donkey anti-rabbit IgG (1/500; Invitrogen) for 1 hour at room temperature. Nuclei were labeled with 500 nM 4’, 6-diamidino-2-phenylindole (DAPI; Invitrogen).

### Statistics

Unpaired nonparametric t-tests were used to evaluate statistical significance at p ≤ 0.05 (GraphPad Prism statistics software, version 6). All quantitative and qualitative analyses are representative of n ≥ 3 biological repeats for all experiments.

## Results and discussion

Lamellar bodies in AT2 cells are members of a subclass of acidic lysosome-related organelles referred to as secretory lysosomes. The acidic pH of lysosomes allows for their specific staining with the basic membrane-permeant fluorophore, LysoTracker Green DND-26 (Ex/Em: 504/511 nm), which selectively accumulates in acidic organelles in live cells. That means that the amount of fluorescence obtained from staining with LysoTracker is directly related to the volume of lysosome-related organelles in a cell. The aim of this study was to use the selective accumulation of LysoTracker in lamellar bodies as a method for subsetting viable AT2 cells by flow cytometry, and tracking their differentiation in live-cell culture by microscopy.

**Figure 1:**
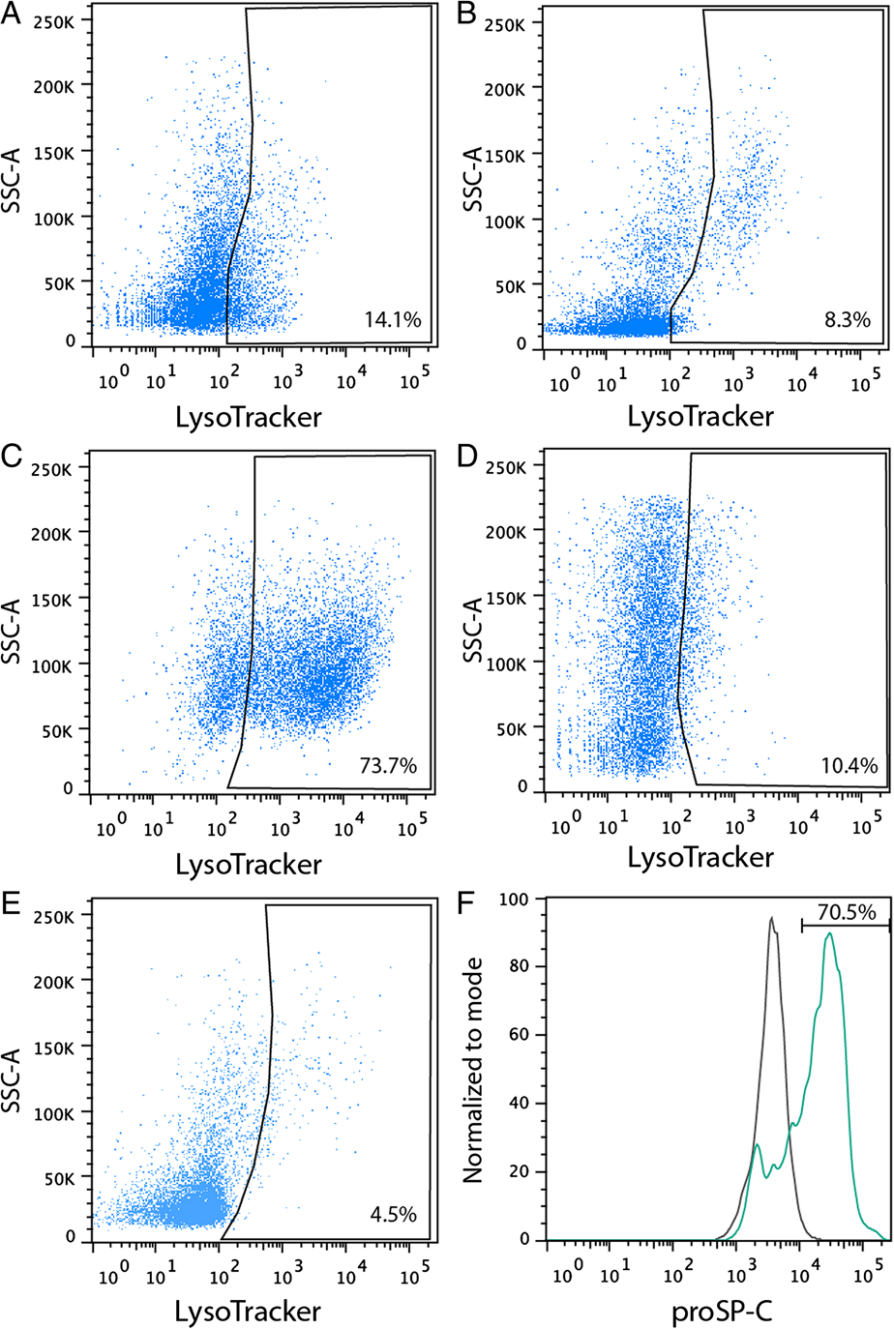
**Subsetting of mouse lung cells with LysoTracker Green DND-26 by flow cytometry.** FACS plots showing LysoTracker staining of single viable **A)** CD45^pos^ CD31^neg^ EpCAM^neg^ cells, **B)** CD45^neg^ CD31^pos^ EpCAM^neg^ cells, **C)** CD45^neg^ CD31^neg^ EpCAM^pos^ cells and **D)** CD45^neg^ CD31^neg^ EpCAM^neg^ cells and **E)** non-viable PI^pos^ cells. Gates represent LysoTracker^pos^ staining based on unstained controls (Additional file 1: Figure S1). **F)** Histogram of proSP-C (green) staining of EpCAM^pos^ lung epithelial cells, compared to Isotype control (grey).

Staining of whole lung cell suspensions with LysoTracker (100 nM, 30 min, 37°C) resulted in 9.8% of single lung cells being LysoTracker-positive. When used in combination with lineage specific antibodies, LysoTracker resolves 14.1% of viable hematopoietic cells (CD45^pos^ CD31^neg^ EpCAM^neg^ Figure 1A), 8.3% of viable endothelial cells (CD45^neg^ CD31^pos^ EpCAM^neg^ Figure 1B), 73.7% of viable epithelial cells (CD45^neg^ CD31^neg^ EpCAM^pos^ Figure 1C) and 10.4% of other viable lineage-negative cells (CD45^neg^ CD31^neg^ EpCAM^neg^ Figure 1D) in the mouse lung. Of these cell populations, epithelial cells exhibited the highest level of LysoTracker staining. In addition, a small fraction (4.5%) of dead or dying cells (PI^pos^) were positive for LysoTracker (Figure 1E). The intensity of LysoTracker staining in epithelial cells is indicative of the labeling of lamellar bodies in AT2 cells. Lamellar bodies are one of the largest lysosomal-related organelles in any cell type and have the capacity to accumulate large amounts of LysoTracker. Although co-localisation of LysoTracker dye and the AT2-specific surfactant protein-C (SP-C) cannot be determined because LysoTracker is non-fixable, the proportion of EpCAM^pos^ LysoTracker^pos^ lung epithelial cells (73.1%) and EpCAM^pos^ SP-C^pos^ cells (70.5%) is comparable (Figure 1F), suggesting that LysoTracker can be used to selectively identify and isolate AT2 epithelial cells by flow cytometry.

**Figure 2:**
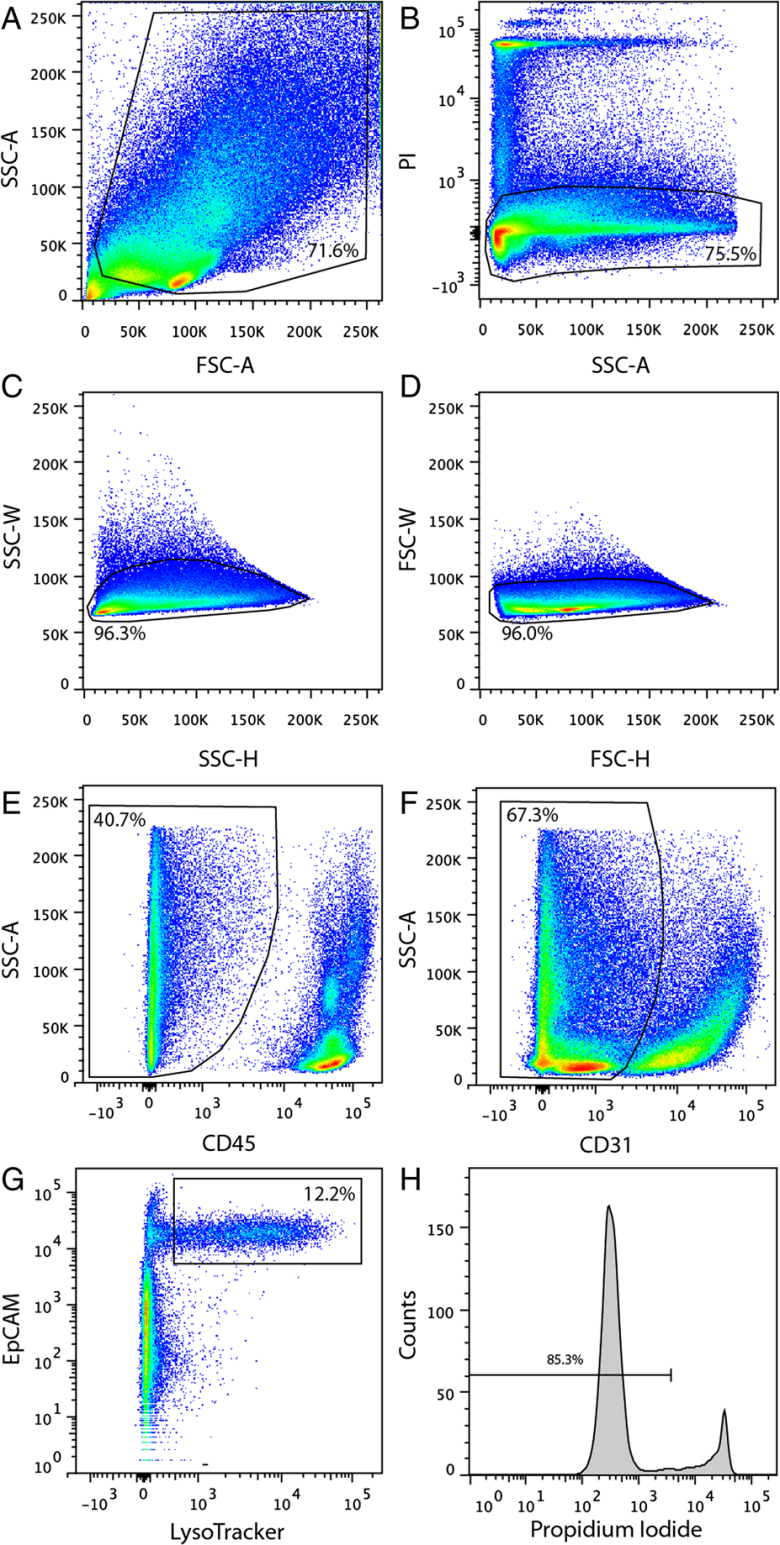
**Isolation of LysoTracker**^**pos **^**alveolar type II cells.** FACS plots showing the sequential gating strategy used to isolate AT2 cells. AT2 cells were gated on the basis of **A)** size (FSC vs. SSC), **B)** viability (PI^neg^) and the exclusion of **C-D)** cell doublets. Viable **E)** non-haematopoietic cells (CD45^neg^) and **F)** non-endothelial cells (CD31^neg^) were then gated on prior to sorting AT2 cells on the basis of their differential expression of **G)** EpCAM and Lysotracker staining. **H)** Histogram showing viability analysis (PI) of sorted EpCAM^pos^ LysoTracker^pos^ cells at 4 hours post-sort.

Here we also show that, when used in combination with CD45, CD31, EpCAM, and PI, the selective uptake of Lysotracker resolves a subset of lung epithelial cells which represents 12.2% of viable (PI^neg^), non-haematopoietic (CD45neg) and non-endothelial (CD31^neg^) lung cells (Figure 2). This is consistent with previous studies reporting that AT2 cells comprise approximately 15% of CD45^neg^ CD31^neg^ cells in the lung [13]. The viability of CD45^neg^CD31^neg^EpCAM^pos^LysoTracker^pos^ cells was assessed at 4 hours post-sorting by staining unfixed cells with PI. The uptake of PI in only 14.7% of cells indicates that 85.3% of cells remained viable after sorting (Figure 2H).

**Figure 3:**
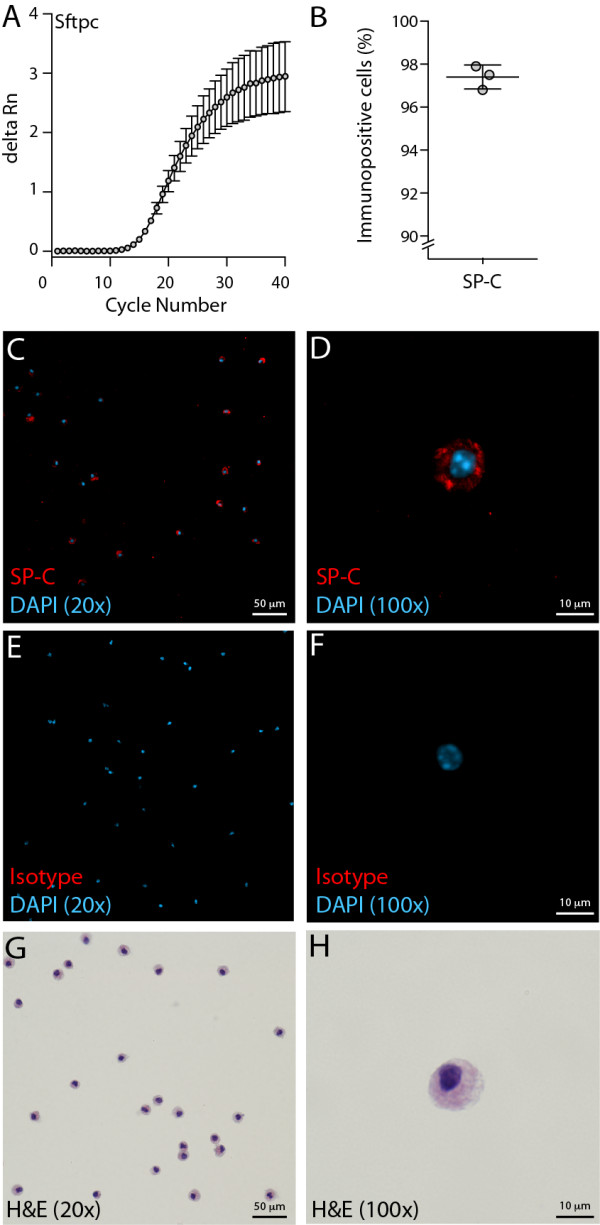
**AT2 lineage analysis of primary PI**^**neg **^**CD45**^**neg **^**CD31**^**neg **^**EpCAM**^**pos **^**LysoTracker**^**pos **^**cells. A)** Amplification plot from qRT-PCR of Sftpc expression in LysoTracker^pos^ AT2 cells. **B)** Percentage of LysoTracker^pos^ AT2 cells that were immunopositive for proSP-C (mean ± SEM, n = 3 independent sorts). Representative images of LysoTracker^pos^ AT2 cells stained with **C-D)** proSP-C (plus DAPI), **E-F)** Isotype control (plus DAPI) and **G-H)** hematoxylin and eosin (H&E).

The presence of AT2 cells in the sorted CD45^neg^CD31^neg^EpCAM^pos^LysoTracker^pos^ cells was confirmed by qRT-PCR, showing robust expression of Sftpc (Ct = 15.84 ± 0.18), which encodes SP-C (Figure 3A). This was supported by immunofluorescent staining confirming the protein expression of SP-C in 97.4 ± 0.3% of the sorted cells (Figure 3B-F). When stained with hematoxylin and eosin, sorted cells exhibited typical cuboidal AT2 cell morphology with large nuclei and cytoplasmic vacuoles (Figure 3G and H). Together this data demonstrates that LysoTracker enriches for a highly pure population of SP-C^pos^ AT2 lung epithelial cells.

A number of transgenic mice that express fluorescent proteins under the control of an SP-C promoter have been useful for analyzing AT2 cells *in vivo*[8,14]. However, recent studies have shown that SP-C is also expressed in other bronchiolar cells including bronchioalveolar stem cells [8,15-18]. The advantage of using LysoTracker to identify AT2 cells is that it specifically marks lamellar bodies, which are restricted to functionally differentiated AT2 cells [13].

The use of LysoTracker as an effective label to isolate AT2 cells for subsequent functional analysis, requires that its uptake into lamellar bodies is not toxic and does not alter cell function. Previous studies showing that the uptake and release of LysoTracker by lamellar bodies recapitulates the process of surfactant storage and secretion by exocytosis in cultured AT2 cells [9,19], suggests that LysoTracker does not affect the functional activity of AT2 cells.

**Figure 4:**
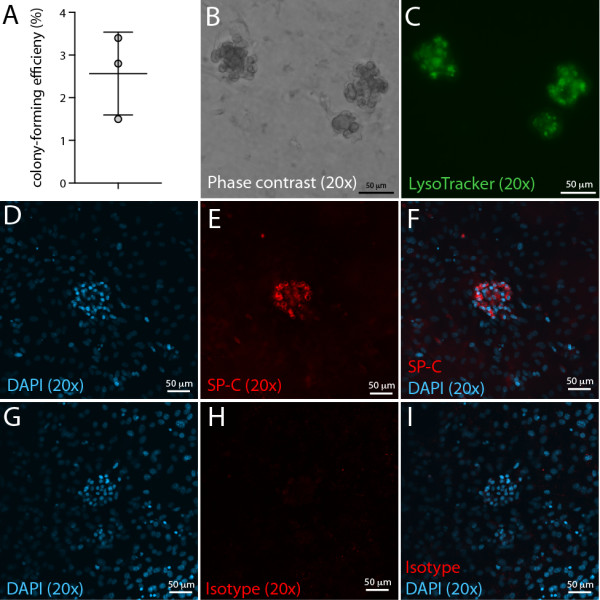
**Colony-forming potential of primary LysoTracker**^**pos **^**AT2 cells. A)** Graph showing colony-forming efficiency of LysoTracker^pos^ AT2 cells cultured in the CFU-Epi assay (mean ± SEM, n = 3). Representative images of AT2 colonies with **B)** Phase-contrast **C)** LysoTracker staining (50 nM for 30 min), **D-F)** proSP-C staining (DAPI, SP-C and Overlay) and **G-I)** Isotype controls (DAPI, Isotype and Overlay).

Several studies have shown that co-culture of epithelial cells with lung mesenchymal stromal cells in the lung epithelial colony-forming unit assay (CFU-Epi) assay stimulates the proliferation and differentiation of lung epithelial progenitor cells, including AT2 cells [8,11,14]. Here we show that AT2 cells sorted on the basis of their CD45^neg^ CD31^neg^ EpCAM^pos^ LysoTracker^pos^ signature profile formed AT2 cell colonies with an incidence of 2.6 ± 0.56% in the CFU-Epi assay (Figure 4A). All colonies were positive for SP-C expression and also stained with LysoTracker in culture (Figure 4B-I), thereby confirming their AT2 cell composition. Non-colony-forming AT2 cells also stained positive for SP-C and Lysotracker, suggesting that differentiated non-proliferating AT2 cells also survive in culture long-term (data not shown). The colony-forming efficiency of AT2 cells in this study is comparable to previous studies of clonogenic cells isolated from SP-C reporter mice (2.3%, [14]) or enriched in the CD31^neg^CD45^neg^CD74^pos^ cell fraction (2.6%; [8]). This is in agreement with the emerging concept that only a subset of mature AT2 cells exhibit progenitor cell activity [14,15], and confirms that LysoTracker does not affect the colony-forming potential of AT2 cells.

**Figure 5:**
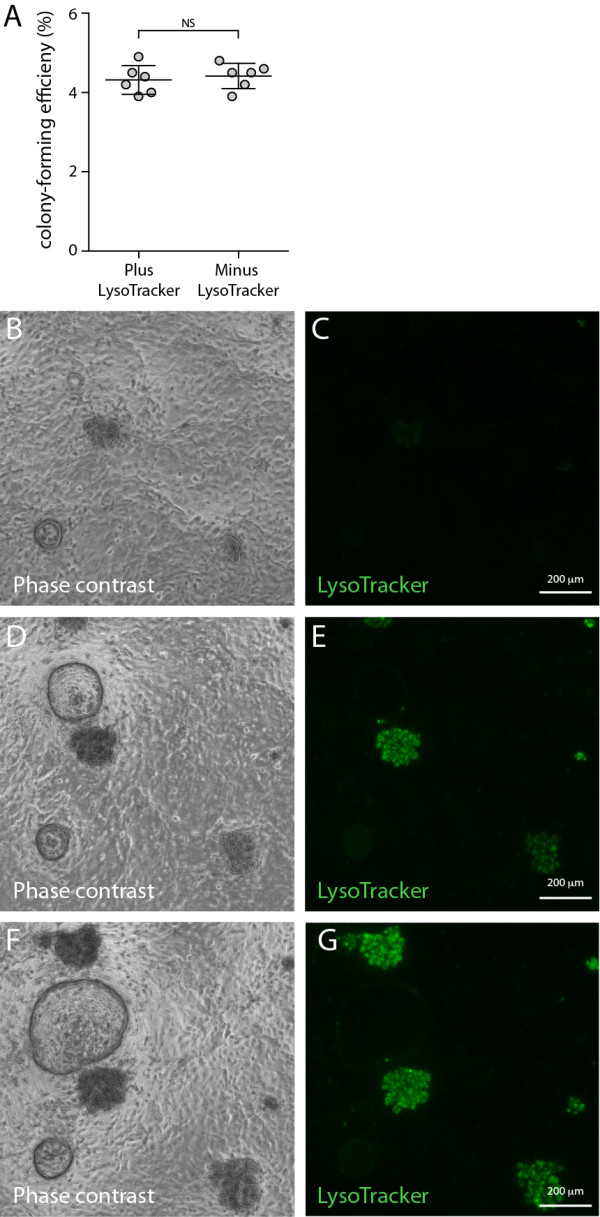
**Live cell imaging of LysoTracker uptake in differentiated AT2 cells *in vitro*. A)** Graph showing the colony-forming efficiency of epithelial stem and progenitor cells (CD45^neg^ CD31^neg^ EpCAM^pos^ CD104^pos^ CD24^low^) cultured in media supplemented with (plus) or without (minus) 50 nM LysoTracker (mean ± SEM, n = 6). Representative phase contrast and fluorescence images of epithelial stem/progenitor-derived colonies in CFU-Epi cultures grown with LysoTracker-supplemented (50 nM) media after **B-C)** 9 days **D-E)** 11 days and **F-G)** 13 days of culture.

Having shown that LysoTracker selectively accumulates in AT2 cells, we next assessed whether this dye could be used to track the differentiation of AT2 cells from stem cells in live-cell culture. Recently, we have shown that lung epithelial stem and progenitor cells (CD45^neg^CD31^neg^EpCAM^pos^CD104^pos^CD24^low^) form colonies of differentiated airway, alveolar or mixed lineage epithelial cells in our CFU-Epi assay which can be identified by their distinct morphologies [11,12]. Importantly, in this study we show that the addition of 50 nM LysoTracker to CFU-Epi culture medium did not affect the growth of epithelial stem and progenitor cell colonies (4.32 ± 0.15%), compared to controls (4.42 ± 0.13%; Figure 5A). This observation further strengthens our assessment that LysoTracker is non-toxic and demonstrates the potential of LysoTracker-supplemented media for tracking the real-time differentiation of AT2 cells in culture. Figure 5 shows that LysoTracker was selectively taken up by alveolar and mixed lineage epithelial colonies, but not airway colonies or early undifferentiated colonies (Figure 5B-G). Although colocalisation of LysoTracker with differentiated lung epithelial markers could not be demonstrated immunohistochemically because LysoTracker is not fixable, we have previously confirmed the presence of AT2 cells in alveolar and mixed lineage colonies and their absence in airway colonies [11]. Therefore positive LysoTracker staining in differentiated alveolar and mixed lineage colonies suggests that the incorporation of LysoTracker marks the differentiation of AT2 cells in culture, while the absence of LysoTracker staining in colonies of airway and undifferentiated epithelial cells confirms that LysoTracker is not incorporated at detectable levels in non-AT2 epithelial cells.

## Conclusions

This study describes a novel and specific method for isolating AT2 cells from mouse lungs by flow cytometry. Compared to current protocols for isolation of AT2 cells, isolating cells on the basis of LysoTracker staining is quicker and results in very high cell purity and viability. This makes it suitable for isolating cells for functional studies *in vitro*. This study has also identified LysoTracker as a useful tool for tracking AT2 cell differentiation in live-cell culture by microscopy. Using this method, we have shown supporting evidence that a subset of mature, differentiated LysoTracker^pos^ AT2 cells exhibit progenitor cell activity with the capacity for self-renewal *in vitro*.

## Abbreviations

AT1: Alveolar type 1; AT2: Alveolar type II; CFU-Epi: Epithelial colony-forming unit assay; DMEM: Dulbecco’s modified eagle’s medium; EpiSPC: Epithelial stem and progenitor cell; FBS: Fetal bovine serum; HBSS: Hank’s buffered saline solution; ITS: Insulin, transferrin and selenium; PI: Propidium iodide; PSG: Penicillin, streptomycin, glutamax; SP-C: Surfactant protein-C.

## Competing interests

The authors declare that they have no competing interests.

## Authors’ contributions

JV participated in design of all experiments, performed data acquisition and analysis and drafted the manuscript. IB contributed to data analysis and helped write the manuscript. JM conceived of the study, lead the design and coordination of the experimental plan, participated in data acquisition, performed data analysis and helped write the manuscript. All authors read and approved the final manuscript.

## Supplementary Material

Additional file 1: Figure S1 Subsetting of mouse lung cells without LysoTracker staining. FACS plots showing LysoTracker unstained controls of single viable A) CD45^pos^ CD31^neg^ EpCAM^neg^ cells, B) CD45^neg^ CD31^pos^ EpCAM^neg^ cells, C) CD45^neg^ CD31^neg^ EpCAM^pos^ cells and D) CD45^neg^ CD31^neg^ EpCAM^neg^ cells and E) non-viable PI^pos^ cells. Click here for file
