# Supplementary figures and images for: LysoTracker is a marker of differentiated alveolar type II cells
Source: Respir Res. 2013 Nov 11;14(1):123. doi: 10.1186/1465-9921-14-123 (PMC3840660; doi:10.1186/1465-9921-14-123)

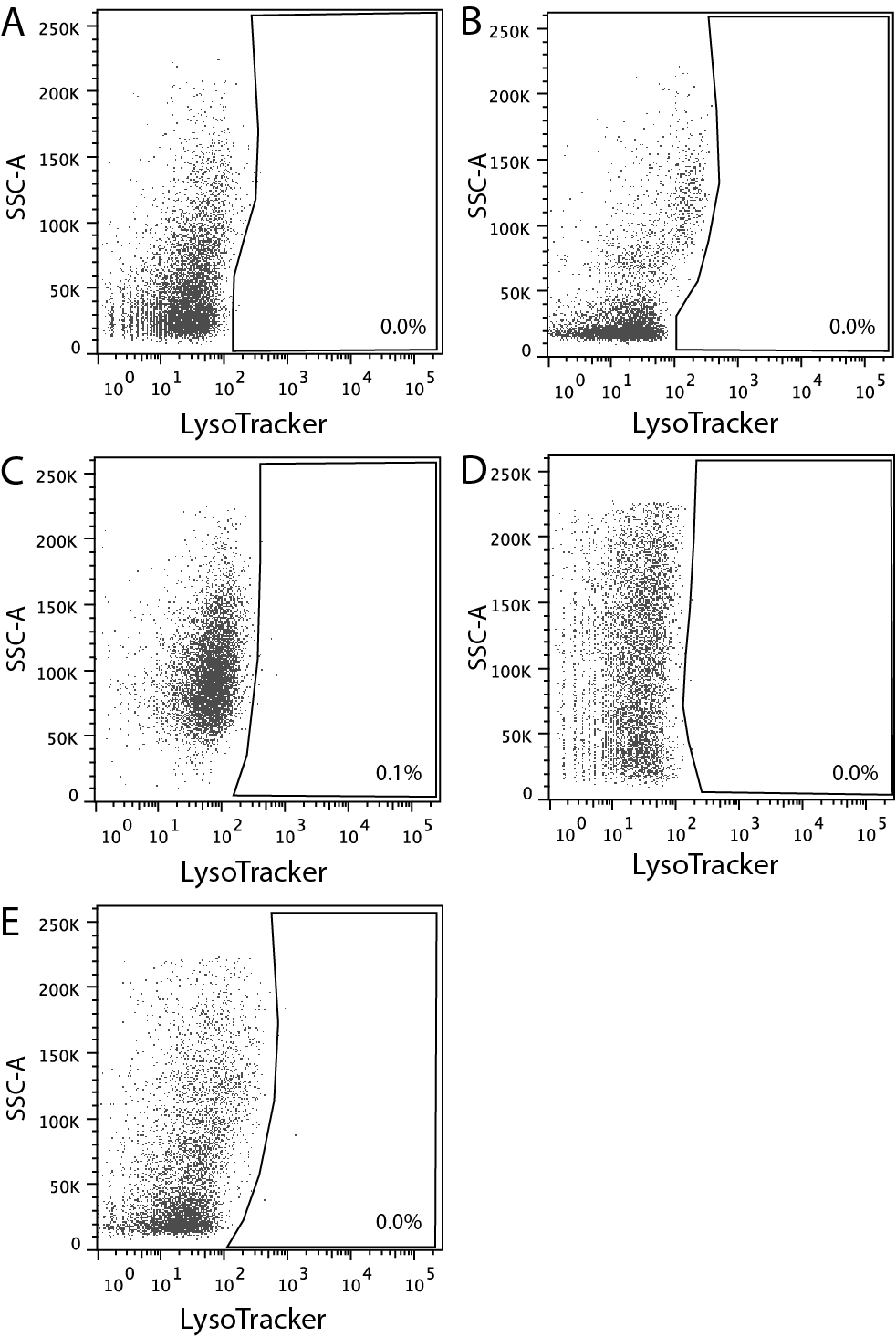

Supplement: Additional file 1: Figure S1 — Subsetting of mouse lung cells without LysoTracker staining. FACS plots showing LysoTracker unstained controls of single viable A) CD45pos CD31neg EpCAMneg cells, B) CD45neg CD31pos EpCAMneg cells, C) CD45neg CD31neg EpCAMpos cells and D) CD45neg CD31neg EpCAMneg cells and E) non-viable PIpos cells. [file 1465-9921-14-123-S1.tiff]
